# Attitudes and Acceptability on HIV Self-testing Among Key Populations: A Literature Review

**DOI:** 10.1007/s10461-015-1097-8

**Published:** 2015-06-09

**Authors:** Carmen Figueroa, Cheryl Johnson, Annette Verster, Rachel Baggaley

**Affiliations:** Escuela Nacional de Salud Pública, Instituto de Salud Carlos III, Madrid, Spain; HIV/AIDS Department, World Health Organization, Geneva, Switzerland

**Keywords:** Key populations, Acceptability, HIV self-testing, Values, Preferences

## Abstract

HIV self-testing (HIVST) is a potential strategy to overcome disparities in access to and uptake of HIV testing, particularly among key populations (KP). A literature review was conducted on the acceptability, values and preferences among KP. Data was analyzed by country income World Bank classification, type of specimen collection, level of support offered and other qualitative aspects. Most studies identified were from high-income countries and among men who have sex with men (MSM) who found HIVST to be acceptable. In general, MSM were interested in HIVST because of its convenient and private nature. However, they had concerns about the lack of counseling, possible user error and accuracy. Data on the values and preferences of other KP groups regarding HIVST is limited. This should be a research priority, as HIVST is likely to become more widely available, including in resource-limited settings.

**Electronic supplementary material:**

The online version of this article (doi:10.1007/s10461-015-1097-8) contains supplementary material, which is available to authorized users.

## Resumen

Autoexaminarse para el VIH con una prueba casera, podría reducir las disparidades del acceso al diagnóstico del VIH, especialmente entre grupos de población claves. Revisamos la literatura disponible sobre la aceptabilidad, los valores y preferencias de la prueba casera en estos grupos de población. Analizamos los datos según el ingreso del país utilizando la clasificación del Banco Mundial, el tipo de muestra, la supervisión ofrecida y otros aspectos cualitativos. La mayoría de los estudios identificados fueron en países con ingresos elevados y con hombres que tienen sexo con hombres (HSH), quienes reportaron una alta aceptabilidad de la prueba casera, debido a su practicidad y privacidad; aunque les preocupaba la falta de asesoramiento, el posible error de usuario y la precisión de la prueba. Existe poca información sobre los valores y preferencias acerca de la prueba casera en otros grupos de población vulnerable. Considerando el aumento de su disponibilidad, incluso en países con pocos recursos, debería ser un área prioritaria en la investigación.

## Introduction

Key populations (KP) (men who have sex with men (MSM), sex workers (SW), people who inject drugs (PWID), transgender people and people in prisons or closed settings) are disproportionately affected by HIV. Pooled HIV prevalence is 10–50 times greater than in general populations [1–4]. Every year there are over two million new HIV infections worldwide, and it is estimated that 40 % of all new adult HIV infections are among KP [5, 6]. Despite such high HIV burden and the increasing global coverage of HIV testing and treatment services, KP remain underserved [5].

Present disparities in access to HIV services among KP are significant. According to recent surveys, nearly 20 % of MSM report that they are “afraid to access health services” and 1 in 10 do not have access to prevention services, including condoms [7]. Regional reports suggest that across 35 countries in sub-Saharan Africa only 60 % of sex workers have received an HIV test in past 12 months, although this may be an over estimation because of non-representative convenience sampling in many instances [8]. In the USA, an estimated 49 % of PWID have received an HIV test in past 12 months [9]. Reaching UNAIDS’ “90 90 90” targets, 90 % of people with HIV knowing their status, 90 % linked to anti-retroviral therapy (ART) and 90 % virally suppressed [10] will not be possible without increased efforts to improve access to and uptake of HIV testing among KP.

HIV self-testing (HIVST) is an emerging approach with the potential to be high impact, low cost and empowering for those who may not otherwise test, particularly among KP. In order to suit a local context, HIVST may be delivered in multiple ways which vary as to type of support, range of access and site of sale or distribution. Although HIVST does not provide a HIV diagnosis, and all reactive self-test results must be confirmed according to national testing algorithms [11], it may stimulate demand for and increase uptake of HIV testing and counseling among KP, who may be more reluctant to or unable to seek existing services.

Several countries have already introduced or are considering the introduction of HIVST as part of national strategic plans, testing strategies and policy and regulatory frameworks [11–14]. At this time, however no optimal approach has been identified, particularly to reach KP [11].

Potential benefits of HIVST among KP identified in the literature include: the possibility to increase access to HIV testing [15, 16], reduce sexual risk behavior [17], and that it may lead to cost-savings in the context of pre-exposure prophylaxis (PrEP) implementation projects [18]. However there are concerns about linkage to further HIV testing and diagnosis, prevention, care and treatment as appropriate to a client’s HIV status, particularly in legally constrained settings, social and emotional harm following HIVST, use for “point-of-sex testing”(where individuals use HIVST to “screen” potential sex partners), risk of sexual disinhibition, or substitution of highly accurate facility-based HIV testing among high incidence populations [19, 20]. Additionally, there are concerns about the potential for coercion to test, for example for SW being forced to test by brothel owners and clients [21, 22].

While there are several systematic reviews highlighting the high acceptability of HIVST [23–25], none focus on KP values and preferences. In July 2014, the World Health Organization (WHO) issued the first consolidated guidelines on HIV prevention, diagnosis and treatment for the five KP groups [26]. This guidance in particular, calls for service delivery approaches that are acceptable and appealing to KP and that will also reduce disparities in coverage and access to HIV services [26]. Based on promising evidence, a changing policy environment, and renewed global emphasis to reach KP and global targets that aim to close the testing gap [10], this review focuses on the acceptability, values and preferences of KP on HIVST.

## Methods

**Fig. 1:**
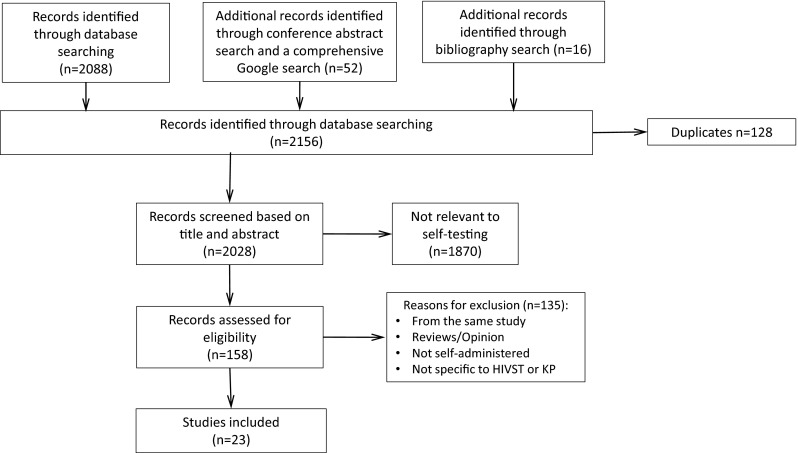
Selection of studies

From April to July 2014 we performed a systematic search to identify evidence on acceptability, values and preferences regarding HIVST among KP (defined as MSM, SW, transgender people, PWID and people in prison). We searched five electronic databases (PubMed, PopLine, Scopus, EMBASE and PsycINFO) and five major HIV/AIDS conference databases (British HIV/AIDS Association, Conference on Retroviruses and Opportunistic Infections, European AIDS Society Conference, International AIDS Society and US National HIV Prevention Conference) for publications between January 1995 and July 2014. Abstracts were included if full-texts were not available. Gray literature was identified through a comprehensive Google search. References were also manually searched to identify other sources. Experts and authors of pertinent studies were contacted for any further references and clarifications (Fig. 1). The search was conducted according to the PRISMA checklist (see Electronic Supplementary Material).

Search terms included ((HIV OR HIV seropositivity OR HIV infections) AND ((self test*) OR (home*test*) OR (rapid*test*))). The search was restricted to human subjects. No language or geographic limitations were placed on the search. Two reviewers screened studies. The first reviewer read study titles and abstracts meeting the inclusion criteria. The second reviewer evaluated the screening criteria and approved selected studies. Disagreements between reviewers were resolved through discussion and consensus. Studies were only included if they used original data, included at least one of the five KP groups, used qualitative and/or quantitative methods that evaluated any aspect on HIVST values and preferences. All other articles were excluded. Studies examining home specimen collection kits were excluded, because participants did not interpret their test result (Fig. 1). Literature was summarized qualitatively according to study design and methodology, location, resource and population.

### Analysis

Documents were analyzed manually through describing their content. Using Microsoft Excel, a systematic framework and extraction tool was developed, to obtain particular information on HIVST values and preferences. After data was extracted it was coded by country income according to the World Bank [27], the educational level (college, high school, elementary or less), the type of specimen collection (oral fluid-based, blood-based, or not specified), KP group (MSM, SW, PWID, transgender people, or people in prison) and the type of support provided (supervised, unsupervised, or not specified).

Values and preferences were defined as participants’ views on HIVST, concerns about HIVST, willingness to pay or buy a HIV self-test, a test kit either specifically packaged for HIVST or a rapid diagnostic test (RDT) distributed or used for HIVST, and other qualitative values and preferences reported by participants. In addition, we examined the acceptability of HIVST, defined as the willingness to take a test in the future or as an increased frequency of testing with a HIV home-test. Reported acceptability was then categorized as high (≥67 %), moderate (66–34 %) or low (≤33 %).

Approaches to HIVST were defined in accordance to the 2014 WHO and UNAIDS technical update on HIVST [28]. Supervised approaches were defined as those which involved direct support from a health worker or a volunteer before or after individuals tested him or herself. Unsupervised approaches were defined as situations when HIVST offered without requiring direct support, but could include the provision of information about where or how to access support services. Studies with no information or comparing types of approaches or specimen collection were analyzed separately. The studies reviewed included both those where participants were able to perform home tests, and those which did not include self-tests but explored survey participants’ values and preferences.

We examined the process of linkage within HIVST for studies where HIVST was performed and where HIVST was not performed by participants answering a questionnaire about HIVST. We primarily analyzed linkage in any study reporting linkage from HIVST to further HIV testing, to receiving a HIV diagnosis in a facility, and/or to enrolment in HIV prevention, care or treatment services. As a secondary analysis we also examined studies which reported on participants’ “intention to link” following a reactive HIV self-test result.

### Quality Assessment

A quality critique of quantitative data from cross-sectional (Electronic Supplementary Tables S1, S2) and cohort studies (Electronic Supplementary Table S3) was performed using the STROBE checklist [29]. Reports were critiqued using the STROBE checklist as they were reporting outcomes of a cross-sectional study [30, 31]. For a conference abstract reporting a randomized control trial [16] (Electronic Supplementary Table S4) we used the CONSORT guidelines [32]. Qualitative studies [17, 31, 33–35] were evaluated with a guide for critically appraising qualitative research [36]. Due to lack of standardized reporting of primary and secondary outcomes, and heterogeneity of data on values and preferences, a meta-analysis was not conducted.

## Results

**Fig. 2:**
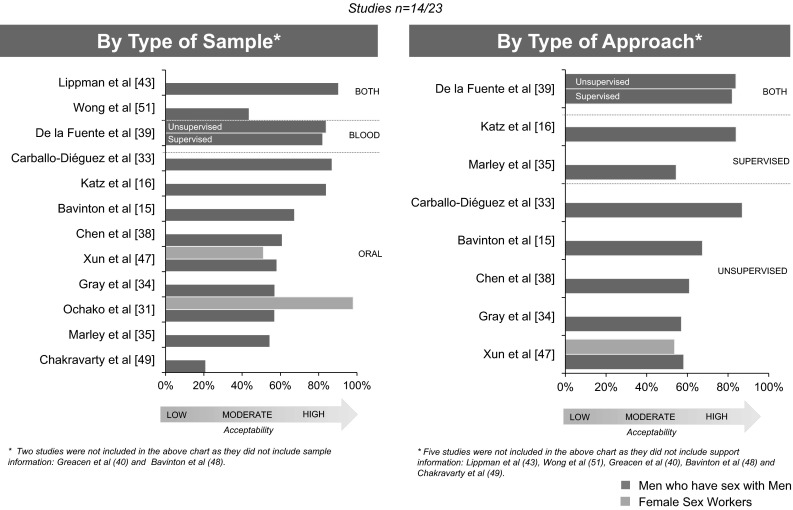
Studies evaluating HIV self-testing acceptability

We identified 2156 citations from databases, abstracts and bibliography searches, after removing duplicates and irrelevant articles (Fig. 1). After an initial screening, we retrieved 158 citations, following which we removed 135 references that did not pertain to HIVST or KP, or were reviews using data from other studies. Ultimately, 23 studies met our inclusion criteria and were analyzed for this review: 16 (69.6 %) were peer-reviewed articles [15, 17, 33–35, 37–47], five (21.7 %) were abstracts [16, 48–51] and two (8.7 %) were reports [30, 31]. Table 1 presents the characteristics of the 23 included studies. All studies reported on values and preferences on HIVST (Tables 2-3) and 14 studies reported also on acceptability (Fig. 2).

**Table 1:** Characteristics of included studies

No.	Author and year	Setting	Sample size	Type of approach	Type of test	Performed HIVST	Study design	Key populations (%)	Median or mean age (SD or IQR)	Summary score for quality critique^a^
1	Xun (2013) [47]	China	1137	Unsupervised	Oral fluid-based	Yes	Quantitative cross-sectional	MSM (32.6 %) FSW (35.6 %) VCT (31.8 %)	MSM: 26 years (IQR 23–31) FSW: 25 years (IQR 23–28)	66 % (21/32)
2	Carballo-Diéguez (2012) [33]	USA	57	Unsupervised	Oral fluid–based	Yes	Quantitative and qualitative cross-sectional	MSM (100 %)	34.3 years (SD 11.9)	
3	MiraTess (2008) [30]	Netherlands, Germany, United Kingdom, Austria, Switzerland and Belgium	1122	Unsupervised	Blood-based	Yes	Quantitative survey	MSM (36 %) Women and HTX men (64 %)	n/a (IQR 13–76)	47 % (15/32
4	Marley (2014) [35]	China	800	Supervised	Oral fluid-based	Yes	Quantitative and qualitative cross-sectional	MSM (46.3 %) FSW (25 %) VCT(28.6 %)	n/a	66 % (21/32)
5	Ng (2013) [44]	Singapore	994	Supervised	Oral fluid-based	Yes	Quantitative cross-sectional	MSM (16 %) HTX men or women (84 %)	32.4 years (IQR 27.1–40.5)	66 % (21/32)
6	Katz (2012) [16]	USA	133	Supervised	Oral fluid-based	Yes	Randomized control trial	MSM (100 %)	39 years (IQR 30–48)	59 % (10/17)
7	Carballo-Diéguez (2012) [17]	USA	27	Supervised	Oral fluid-based	Yes	Quantitative and qualitative cross-sectional	MSM (100 %)	34 years (SD 11.4)	
8	Mayer (2014) [51]	USA	161	Supervised	Blood-based	Yes	Quantitative cohort study	MSM (97.5 %) TG (2.5 %)	36.5 years (SD n/a)	36 % (4/11)
9	De la Fuente (2012) [39]	Spain	519	Supervised and Unsupervised	Blood-based	Yes	Quantitative cross-sectional	MSM (36.7 %)	n/a*	56 % (18/32)
10	Lee (2007) [42]	Singapore	350	Supervised	Blood-based	Yes	Quantitative cross-sectional	MSM (10 %) HTX men or women (90 %)	33 years (IQR 27–41)	69 % (22/32)
11	Han (2014) [41]	China	1342	Unsupervised	Oral fluid-based and blood-based	No	Quantitative survey	MSM (100 %)	n/a*	66 % (21/32)
12	Spielberg (2003) [46]	USA	460	Unsupervised	Oral fluid-based	No	Quantitative survey	MSM (33.9 %) PWID (24.3 %) HTX men or women and lesbians (41.8 %)	n/a*	63 % (20/32)
13	Bavinton (2013) [15]	Australia	2018	Unsupervised	Oral fluid-based	No		MSM (100 %)	34.3 years (SD 11.5)	63 % (20/32)
14	Gray (2013) [34]	Australia	233	Unsupervised	Oral fluid-based	No	Quantitative and qualitative cross-sectional	MSM (96.1 %) HIV non-positive or not aware (3.9 %)	38.6 years (SD n/a)	59 % (19/32)
15	Skolnik (2001) [45]	USA	134	Unsupervised	Blood-based	No	Quantitative survey	MSM (45 %) HTX men or women and Bisexual women or lesbians (55 %)	n/a (IQR 18–59)	56 % (18/32)
16	Chen (2010) [38]	Australia	172	Unsupervised	Oral fluid-based	No	Quantitative cross-sectional	MSM (100 %)	32 years (IQR 15–71)	56 % (18/32)
17	Ochako (2014) [31]	Kenya	982	n/a	Oral fluid-based	No	Quantitative and qualitative cross-sectional	MSM (10.2 %) FSW (10.2 %) GP (79.6 %)	MSM: 24 years (IQR 18–49) FSW: 26 years (IQR 18–49) GP: 27 years (IQR 18–49)	72 % (23/32)
18	Lippman (2014) [43]	Brazil	356	n/a	Oral fluid-based and blood-based	No	Quantitative survey	MSM (100 %)	26 years (IQR 22–33)	63 % (20/32)
19	Bilardi (2013) [37]	Australia	31	Supervised	Oral fluid-based	No	Qualitative description	MSM (100 %)	n/a*	n/a
20	Chakravarty (2014) [50]	USA	310 couples	Supervised	Oral fluid-based	No	Quantitative cohort study	MSM (100 %)	43.1 years (IQR n/a)	45 % (5/11)
21	Wong (2014) [52]	Hong Kong SAR, China	1122	n/a	Oral fluid-based and blood-based	No	Quantitative cross-sectional	MSM (100 %)	n/a	73 % (8/11)
22	Greacen (2013) [40]	France	5908	n/a	n/a	No	Quantitative survey	MSM (100 %)	35 years (IQR 27–43)	59 % (19/32)
23	Bavinton (2014) [48]	Australia	567	n/a	n/a	No	Quantitative survey	MSM (87.1 %) non-HIV-positive men (12.9 %)	38.5 years (SD n/a)	54 % (6/11)

*HIVST* HIV self-testing, *n/a* not available, *MSM* Men who have sex with men, *HTX* Heterosexual, *FSW* female sex workers, *TG* transgender people, *VCT* voluntary counselling testing, *GP* general population, *IQR* interquartile range, *SD* standard deviation

* Age reported as a percentage

^a^The summary score for quality critique represents the number of criteria reported over the total number of criteria

**Table 2:** Values and preferences of studies with supervised support

	Low income country	Middle income countries		High income countries				
Study	Ochako et al. [31]^a^	Lippman et al. [43]^a^	Marley et al. [35]	Bilardi et al. [37]	Ng et al. [44]	Katz et al. [16]	Chakravarty et al. [49]^a^	Carballo-Diéguez et al. [17]
Study aims	Identify willingness to use oral fluid-based RDTs for self-testing, and factors associated with the potential adoption and use of oral HIVST	Determine the acceptability of HIVST, compared to clinic-based HIV testing, and explore preferences for HIVST	Assess feasibility and acceptability of oral fluid-based RDTs among MSM, FSW and VCT clients; assess the quality of HIVST with oral fluid-based RDTs compared to VCT and assess attitudes towards HIVST among FSW	Explore the views of MSM on HIVST, including acceptability, potential use, benefits and limitations	Compare user acceptability and feasibility on HIVST using RDTs versus RDTs used at the POC by trained personnel, including user attitudes towards oral fluid-based RDTs used for HIVST	Described ease of use and acceptability of HIVST using oral fluid-based RDT among high risk MSM	Explore the attitudes on HIVST among MSM couples	Assessed whether at-risk HIV-uninfected MSM would use HIVST to screen potential sexual partners prior to intercourse
Participants pros’	MSM: 70 % easy to use; 68 % guarantees confidentiality and privacy; 28 % required no visit to a health facility; 21 % saves times; and 12 % convenient* FSW: 70 % guarantees confidentiality and privacy; 52 % easy to use; 32 % convenient; and 23 % required no visit to a health facility*	68 % (244/356) Privacy	FSW: 96.5 % (193/200) convenient, 95.5 % (191/200) painless, 13 % (26/200) easy to use and 14 % (28/200) privacy	Convenience, privacy, painless, and easy to use*	95 % Convenience*	63.2 % Easy to use*	56 % Convenience*	Convenience*
Concerns	MSM: 44 % (n/a) were afraid of a positive result. FSW: 3 % (3/100) were afraid of a positive result, 1 % (1/100) afraid of misinterpreting the results, and 1 % (1/100) believed health workers should perform the test	30.6 % (109/356) User error and 22 % (79/356) lack of counseling	FSW: 55.5 % (111/200) accuracy	Lack of counseling, accuracy*	n/a	n/a	Confidentiality and lack of time*	User error*
Preferences	MSM: 56 % would procure and perform the test on their own; 49 % preferred to obtain the test kits in either private chemists/pharmacies or 47 % in government clinics* FSW: 95 % would procure and perform the test on their own; 75 % preferred to obtain the kits from private chemists/pharmacies, 53 % in government facilities and 13 % in supermarkets/shops*	47 % (167/356) preferred HIVST over testing in clinics; 60 % (213/356) would HIVST to make choices about unprotected sex with regular partners and 52 % (184/356) with new partners	FSW: 42.8 % (83/200) preferred saliva testing, while 57.2 % (111/200) still preferred blood testing; 7.5 % (5/200) wanted simplified procedure and 7 % (14/200) wanted the test to be offered free	Available OTC and online, provide access to 24 h counselling and with proper instructions*	88.9 % (884/994) available OTC, 88.6 % (881/994) prefer to do it in private and 73.9 % (735/994) felt that post-test counseling was necessary	n/a	n/a	Available as OTC*
Willingness to pay (US$)	Range in study $ 0.54–4.35 MSM: 57 % would be willing to pay. Mean max price $ 3.35 FSW: 94 % would be willing to pay. Mean max price $ 3.1	n/a	n/a	In average $ 9.2–18.5	28 % (277/994) Would pay at least $ 15	46 % Would pay ≤ $ 20 26 % would pay ≥ $ 40	n/a	n/a
Serious adverse self testing events	n/a	n/a	n/a	n/a	n/a	n/a	n/a	n/a
Linkage to care	MSM:50 % would seek post-test counseling and confirmation of results* FSW: 75 % would go to a health facility/VCT for confirmation*	n/a	n/a	n/a	n/a	2 HIV reactive tests: [1] search confirmatory testing and care immediately [2] search confirmatory testing and care after 2 months	n/a	n/a

	High income countries					
Study	Mayer et al. [50]	De la Fuente et al. [39]^b^	Lee et al. [42]	Wong et al. [51]^c^	Greacen et al. [40]^c^	Bavinton et al. [48]^c^
Study aims	Assessed the feasibility and acceptability of biweekly HIVST at home using whole blood-based/fingerstick RDTs	Evaluate the feasibility of HIVST including obtaining the sample and interpreting results (not their own)	Compare user acceptability and feasibility of using RDTs for HIVST versus RDTs by trained providers at the POC	Describe the patterns of HIVST users among MSM	Estimate the proportion of MSM interested in authorized kits for HIVST, their reasons for being interested and their correlates	Explore the motivations of using and implications of using HIVST
Participants pros’	n/a	n/a	88 % (300/350) Easy to use	50 % Easy to use, 41.2 % convenience, 25 % privacy*	23 % Convenience and 17 % privacy*	47.6 % Privacy*
Concerns	n/a	n/a	n/a	n/a	6 % Accuracy, 6.1 % lack of counselling and 3.6 % of user error	n/a
Preferences	56.5 % preferred HIV testing at home, and 23.6 % preferred testing in a doctor’s office. 90.0 % would be comfortable testing partners at home*	n/a	88 % (304/350) Thought the kit should be sold in public outlets. 89 % (307/350) preferred to take the test in private; 87 % (296/350) thought counselling is needed before testing	16.2 % Didn’t want counselling*	n/a	n/a
Willingness to pay (US$)	n/a	87.3 % Were willing to pay $ 1.25–49 and 5.2 % were reluctant to pay*	Between $ 7 and $ 13 (n/a)	n/a	n/a	n/a
Serious adverse self testing events	n/a	n/a	n/a	n/a	n/a	n/a
Linkage to care	Two participants became HIV infected for an annualized incidence of 3.86 (0.47–19.74). Both were linked to care	n/a	n/a	81.6 % believed that they would get timely treatment if infected with the virus*	n/a	n/a

*FSW* female sex workers, *RT* rapid testing, *OTC* over-the-counter, *HIVST* HIV self-testing, *n/a* not available, *MSM* Men who have sex with men, *VCT* voluntary counselling testing, *POC* point of care

* Percentage or raw number not available

^a^Type of approach non available

^b^Both types of support: supervised and unsupervised

^c^Support non available

**Table 3:** Values and preferences of studies with unsupervised support

	Middle income countries		High income countries						
Study	Xun et al. [47]	Han et al. [41]	Spielberg et al. [46]	Bavinton et al. [15]	Carballo-Diéguez et al. [33]	Gray et al. [34]	Skolnik et al. [45]	Chen et al. [38]	MiraTess [30]
Study aims	Assess the willingness to accept the oral fluid HIV rapid testing and its associated factors among most-at-risk populations	Examines the frequency and the correlates of HIVST among MSM	Determine strategies to overcome barriers to HIV testing among persons at risk	Explore which gay men would increase their frequency of HIVST and examine reasons for not testing among men who have never been tested	Investigate if participants use the HIVST to test themselves/screen sexual partners prior to sexual intercourse and the strategies that they would use	Determine the acceptability and epidemiological impact of increases in HIV testing	Examine preferences for specific types of HIV tests as well as for test attributes such as cost, counselling and privacy	Examine the views of Australian MSM on the acceptability and potential uptake of rapid oral testing for HIV in clinic and home-based settings	Describe the people who prefer to test themselves, reason for testing and their experiences
Participants pros'	MSM: 21 % painless* FSW: 33 % painless*	n/a	Privacy and convenience*	58.7 % (1186/2018) convenience, 75.5 % (1524/2018) immediate results and 42.3 % (854/2018) privacy	n/a	n/a	24.6 % Privacy and 30 % convenience*	39 % Convenience, privacy, painless and easy to use*	53 % Privacy, 46 % easy to use and 31 % convenience*
Concerns	MSM: 49.1 % accuracy and 7.5 % not been free* FSW: 42.2 % accuracy and 9.4 % not been free*	n/a	31 % Had concerns, mostly on accuracy, user error and lack of counseling*	n/a	User error and kits for HIVST not being free*	n/a	n/a	54 % Lack of counseling, accuracy and user error*	n/a
Preferences	n/a	34.7 % Referred to obtain the test on the internet*	n/a	n/a	50 % use it with new partners and preferred oral fluid-based RDTs over fingerstick/whole blood-based RDTs for HIVST*	58.8 % (137/233) preferred oral fluid-based testing and 54.1 % (126/233) finger-prick testing	n/a	n/a	n/a
Willingness to pay (US$)	Median price (IQR) MSM $ 6.5 (3.0.11.3) FSW $ 4.8 (1.6.8.1)	9.3 % paid < $ 8 1.2 % paid > $ 50	Median price (IQR) $ 30 (n/a)	n/a	n/a	n/a	24 % would pay $ 50	n/a	n/a
Serious adverse self testing events	n/a	n/a	n/a	n/a	Intended to coerce someone to test for HIV (1/57)	n/a	n/a	n/a	n/a
Linkage to care	n/a	n/a	n/a	n/a	If self-test result is reactive several participants will seek confirmatory testing followed by treatment*	n/a	n/a	n/a	If HIVST result is reactive 98% will link to care*

*n/a* not available, *MSM* Men who have sex with men, *HTX* heterosexual, *FSW* female sex workers, *VCT* voluntary counseling and testing, *POC* point of care, *PWID* people who inject drugs, *OTC* over-the-counter

* Percentage or raw number not available

One study (4.3 %) was performed in a low-income country (LIC) [31]. Four studies (17.4 %) were performed in middle-income countries (MIC) [35, 41, 43, 47] and 18 studies (78.3 %) were performed in high-income countries (HIC) [15–17, 30, 33, 34, 37–40, 42, 44–46, 48–51]. Age was reported in 21 studies (91 %), and ranged from 13 to 76 years [15–17, 30, 31, 33, 34, 37–50]. Education level was reported in 14 studies (61 %) [15, 17, 31, 33, 34, 39–47]. In 11 studies more than half of the total sample had at least a college education [15, 17, 33, 34, 39–43, 45, 47]. All studies included MSM (100 %) [15–17, 30, 31, 33–35, 37–51], three studies (13 %) included female sex workers (FSW) [31, 35, 47], one study (4.3 %) included PWID [46], one study (4.3 %) included transgender women [50], and no studies included people in prison. Sample size varied from 27 to 5908 participants. Thirteen studies used oral fluid-based HIV RDTs [15–17, 31, 33–35, 37, 38, 44, 46, 47, 49], five used fingerstick/whole blood-based HIV RDTs [30, 39, 42, 45, 50], three used both types of HIV RDTs [41, 43, 51] and two did not provide information on the type of specimen collection used [40, 48]. Nine studies used an unsupervised approach [15, 30, 33, 34, 38, 41, 45–47], seven used a supervised approach [16, 17, 35, 37, 42, 44, 50], six did not report this information [31, 40, 43, 48, 49, 51], and one compared both approaches [39]. In 10 studies participants performed a HIVST RDT (n = 10/23), [16, 17, 30, 33, 35, 39, 42, 44, 47, 50], of which six used a supervised approach [16, 17, 35, 42, 44, 50] and three used an unsupervised approach [30, 33, 47] and one used both [39]. The remainder did not self-test for HIV but were surveyed about their values and preferences (n = 13/23) [15, 31, 34, 37, 38, 40, 41, 43, 45, 46, 48, 49, 51]. Nearly all studies (95.7 %) were observational (14 cross-sectional, one qualitative, two cohort, five mixed method (cross-sectional and qualitative)) [15, 17, 30, 31, 33–35, 37–51] and one study (4.3 %) was a randomized control trial [16] (Table 1).

## Acceptability

Out of 14 studies, eight were consistent with a high acceptability, as defined above [15, 16, 31, 33, 39, 40, 43, 48], five studies with moderate [34, 35, 38, 47, 51] and one study with low acceptability [49]. The acceptability rate ranged from 21 to 98 %. All studies included MSM [15, 16, 31, 33–35, 38–40, 43, 47–49, 51] and three studies included FSW [31, 35, 47]. Chakravarty et al. reported the lowest acceptability, this study was in MSM couples in USA, surveyed about an oral fluid-based HIV RDT, and 21 % of HIV negative men aware of the test were extremely likely to use the test [49].

**Fig. 3:**
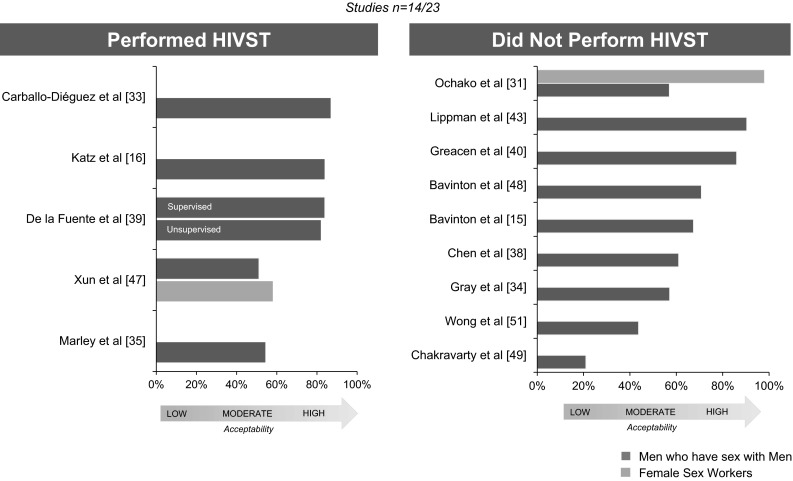
HIV self-testing experience among studies evaluating acceptability

Two studies reported acceptability by KP type [31, 47]. In Kenya, participants where surveyed about an oral fluid-based HIV RDT, and FSW (98 %) reported a higher acceptability than MSM (57 %) [31]. In China, acceptability was very similar between MSM (58.2 %) and FSW (51.1 %), in this study, participants were surveyed also about an oral fluid-based HIV RDT, but 6.9 % had ever taken one before [47] (Figs. 2, 3).

In five studies (n = 5/14) participants self-administered an HIV RDT, but did not necessarily interpreted their test results (Fig. 3) [16, 33, 35, 39, 47], remainder studies (n = 9/14) participants were surveyed about HIVST [15, 31, 34, 38, 40, 43, 48, 49, 51]. Overall, no large differences in acceptability were identified across type of approach, type of specimen collection, having performed an HIVST, country income, group of KP, or educational level of population.

## Values and Preferences for HIVST

Twenty-three studies assessed key population values and preferences on HIVST (Tables 2, 3).

### Benefits of HIVST

Findings about benefits were variously documented in 18 articles, including: (a) Convenience, (b) Privacy, (c) Painless, and (d) Easiness to Use.

Across reviewed studies convenience (n = 13/18) [15, 17, 30, 31, 35, 37, 38, 40, 44–46, 49, 51] and privacy (n = 12/18) [15, 30, 31, 35, 37, 38, 40, 43, 45, 46, 48, 51] were reported as benefits of HIVST most frequently, followed by easiness-to-use (n = 8/18) [16, 30, 31, 35, 37, 38, 42, 51] and painlessness (n = 4/18) [35, 37, 38, 47]. Ochako et al. reported that in Kenya HIVST is easy to use, even for people with low education [31].

Privacy was more frequently reported as a benefit of HIVST in studies using an unsupervised approach (n = 5/6) [15, 30, 38, 45, 46] compared to those using a supervised approach (n = 2/6) [35, 37]. Although approach was not reported 71 % of MSM in Brazil, reported that HIVST would offer more privacy than HIV testing facilities [43]. In general, the benefits for HIVST described by participants across studies, remain similar; even when analyzed by country income, type of KP, participant education level, type of specimen collection, having performed an HIVST and type of approach.

### Preferences for HIVST Attributes

Twelve articles provided information on KP preferences [17, 31, 33–35, 37, 41–44, 50, 51]. Preferences for test type of sample collection (oral fluid-based or fingerstick/whole blood-based) (n = 7/12), distribution (n = 7/12), instructions (n = 2/12), the availability to link to counseling (n = 4/12), and how they would like to use the test (n = 6/12) were reported. Preferences for HIVST attributes varied across country income setting, type of approach, having performed a self-test for HIV and type of specimen collection. However, in general, participants reported preferring HIVST with an oral fluid-based HIV RDT (n = 4/12), to blood-based HIV RDT (n = 3/12) [33–35, 43].

Five studies from Kenya, Singapore, USA and Australia reported MSM and FSW generally prefer HIVST to be available over-the-counter [17, 31, 37, 42, 44], three of which participants have performed an HIVST [17, 42, 44], and two studies from Australia and China, reported that MSM preferred HIVST to be available through the Internet, in neither of the two MSM participants have performed an HIVST [37, 41]. MSM participants in Australia, desire HIVST to be available over-the-counter, but specifically with proper instructions for use on how to perform a HIV RDT and interpret the test result [37].

Three studies reported participants prefer having counseling available [37, 42, 44]. However, one study in Hong Kong SAR China among MSM reported that 16.2 % of participants prefer HIVST without counseling [51].

### Willingness to Pay

Willingness to pay for a HIVST kit if sold was documented in 11 articles [16, 31, 35, 37, 39, 41, 42, 44–47]. Willingness to pay varied across population, country income settings, type of specimen collection, and type of approach. In HIC settings, study participants were willing to pay between ≤US$20 and ≥US$50 [16, 37, 39, 42, 44–46]. In MIC settings, participants were generally willing to pay between (US$1 to US$20) [41, 47]. A study from China reported that MSM were willing to pay US$6.50 (US$3–US$11), slightly more than FSW who were willing to pay US$5 (US$2–US$8) [47]. In LIC settings, participants were willing to pay between US$0.54–US$4.35 [31]. According to this study in Kenya, MSM were willing to pay (US$3.35), slightly more than FSW who were willing to pay US$3.10 [31].

Participant willingness to pay in all supervised HIVST studies (n = 4/11) ranged between (≥US$1 to ≥US$20) [16, 37, 42, 44]. In 2/11 studies using an unsupervised approach, participants were willing to pay between (>US$20 to ≥US$50) [45, 46]. Reluctance to pay (range 5.2–11 %) was only reported in four studies where MSM and FSW participants have performed an HIVST, these studies examined both approaches and were in MIC and HIC settings [16, 35, 39, 47]; all but one used oral fluid-based HIV RDT [16, 35, 47].

### Reported Concerns of HIVST

Concerns about HIVST were documented in 11 articles [17, 31, 33, 35, 37, 38, 40, 43, 46, 47, 49]. The majority of the studies, in which concerns were reported, stated that participants had concerns about user error (n = 7/11) [17, 31, 33, 38, 40, 43, 46]; followed by low accuracy (n = 6/11) [35, 37, 38, 40, 46, 47], lack of counseling (n = 6/11) [31, 37, 38, 40, 43, 46] and HIVST not being free (n = 2/11) [33, 47].

Concerns were more commonly reported in studies using oral fluid-based RDT (n = 9/11) [17, 31, 33, 35, 37, 38, 46, 47, 49]. Lack of counseling was not a concern in studies where MSM and FSW participants have performed an HIVST [17, 33, 35, 47]. However, concerns for HIVST generally remain the same when analyzed by country income, KP group, participant education level, and type of approach.

### Linkage to Care

Six studies reported on some aspect of linkage to care from HIVST, of which the majority were in HIC settings [16, 30, 31, 33, 50, 51]. Two studies, Katz et al. [16] and Mayer et al. [50] reported actual linkage and enrolment in care following HIVST. Katz et al. [16] reported two participants with reactive self-test results who were diagnosed HIV positive: one participant searched immediately for additional HIV testing and care and the other waited two months before seeking further HIV testing and care [16].

The remainder of the studies reported on “intention to link” following HIVST. In studies from HIC settings, the majority of participants reported that if they received a reactive HIV self-test result they would seek for additional testing and if diagnosed HIV-positive, then  treatment (range 81.6–100 %) [30, 33, 51]. A study in LIC setting reported that 50 % of MSM would seek post-test counseling and confirmation of results and 75 % of FSW stated that they would go to a health facility for confirmation, after self-testing for HIV [31]. Overall, no differences were found when analyzed by test type of specimen collection, educational level, having performed an HIVST and type of approach.

### Adverse Events Resulting from HIVST

There was little information on adverse events reported in reviewed studies. In this review, one study among MSM in the USA, who had performed an oral fluid-based HIV RDT, reported that complicated situations could lead to verbal confrontations or violence among participants who self-tested or proposed self-testing with a sex partner. Also they reported that special circumstances, such as infidelity, could lead to coercively test a partner, a potentially more adverse event [33]. No other serious adverse events were identified.

### Quality of Studies

Quality of studies varied. In general, studies did not report sufficient information about qualitative methods and data collection tool, there was also a lack of compliance on how they assessed and measured the different values and preferences. Qualitative data were sparse and an incomplete reporting of data in abstracts and reports limited the evaluation of quality. This lack of clear evaluation of values and preferences limited our understanding of collected data.

## Discussion

Twenty-three studies reporting acceptability and other values and preferences of KP regarding HIVST were identified. Values and preferences were largely consistent. This may be because many of the included studies had some similar study characteristics. For instance, the majority of included studies were from HIC settings (n = 18/23), among participants with high educational level (n = 11/23), using oral fluid-based RDT (n = 13/23), using unsupervised approaches (n = 9/23), and were almost exclusively among MSM (n = 23/23). Very few studies in this review included FSW, PWID, transgender people (n = 5/23).

Evidence for high acceptability was evident among MSM in HIC settings using oral specimen collection. This aligns with existing literature on HIVST, which suggest users (including the general population) may prefer oral fluid-based HIV RDT to fingerstick/whole blood-based HIV RDT because they are reportedly easier to perform and are perceived to be less painful [52, 53]. Out of all studies reviewed, Chakravarty et al. reported the lowest acceptability of HIVST. However this study only reported acceptability among HIV-negative MSM who were aware of HIVST and reported that they were “extremely likely” to self-test for HIV. Since the study did not report on other levels of acceptability, such as “somewhat likely”, “likely” or “very likely”, we could not infer whether this is reflective of actual acceptability of HIVST among MSM [49].

Research is still ongoing and there are emerging reports from KwaZuluNatal, South Africa which suggest that fingerstick/whole blood-based HIV RDT can also be easy to perform and accurate, when accompanied with clear instructions, packaging and appropriate test system design [54]. In April 2015, two fingerstick/whole blood-based RDTs recently satisfied the legislative requirements in the European Econonomic Area: the BioSure HIV Self Test (BioSure Ltd, UK), sold online at £29.95 [58] and the autotestVIH (Aaz Labs, France) will be sold in pharmacies around 23–28 euros [59]; as an additional option for people to now their HIV serostatus. Various other products are under development and could be adapted for HIVST, including painless or integrated lancets, simplified sampling systems, integrated buffer delivery systems and shorter minimum and maximum reading time [11].

Some studies report that participants desire access to counseling [37, 42, 44], while a study in Hong Kong SAR China with MSM, reported that 16 % preferred HIVST because of the “lack of counseling” [51]. Ways to provide information about or how to link to counseling services, as part of HIVST, should therefore be considered including: face-to-face through community health workers, internet-based, SMS or mobile phones, or computer-based programs.

Studies with unsupervised or an unknown approach to HIVST frequently reported concerns on user error and poor accuracy. These concerns could potentially be overcome by providing links to support and counseling services and clear instructions for use. There might be a small controversy with the benefit of privacy and the concern of an increased user error, depending on the approach, in our findings MSM were not strongly positioned that HIVST has to be performed strictly by a professional [37, 42, 44, 51]. In particular, KP may need more information on how user error can be reduced, accuracy rates and the need for confirmation; especially if HIVST is unsupervised.

Willingness to pay was difficult to compare across all studies, as there were different price points and some used overlapping intervals. Overall willingness to pay was higher in HIC settings [16, 37, 39, 42, 44–46] compared to MIC settings [35, 41, 47] or LIC settings [31], and lower in supervised HIVST [16, 37, 42, 44] than for unsupervised HIVST [41, 47]. This may be because supervised HIVST is viewed as similar to current HIV testing services, which are often free of charge. KP may also be willing to pay more for unsupervised HIVST because it offers greater privacy; which was a key benefit and value of HIVST, reported by KP.

All studies in the USA (reporting willingness to pay between US$1 to ≥US$50) were conducted using oral fluid-based HIV RDT [16, 45, 46], and prior to the US Food and Drug Administration approval of the OraQuick^®^ In-Home HIV Test [55]. Currently, this product retails direct to consumers for US$40 [56]. The studies reviewed suggest that reluctance to pay was only reported in studies were participants have performed an HIVST, also concerns about the cost of HIVST, were both in MIC and HIC settings. Thus, for HIVST to have higher uptake, it will likely need to be subsidized or free of charge to clients. So far a lowest price has been negotiated, for research purposes the professional use version of this test is available in Kenya for approximately US$11 [18] and in Malawi for US$3 [57].

Evidence on linkage to care and treatment among KP is limited and requires further research. Two studies among MSM in the USA reported actual linkage to HIV testing and diagnosis and enrollment in HIV care and treatment [16, 50]. Three studies reported that more than 80 % of participants with a potential or an actual HIV positive test result would seek confirmatory HIV testing and care [16, 30, 51]. Proactive approaches to support the unique needs of KP may be considered and adapted, for example a study in Malawi among general population offering home (ART) assessment found a three-fold increase in linkage to ART, compared to facility-based HIV testing [60]. It is essential that users with a reactive HIV self-test result first link to further testing and receive an HIV diagnosis; and that users also link to HIV prevention, care and treatment services, as appropriate to their HIV status, in a timely manner. Special attention should be paid to additional risks for KP, including young and adolescent KP. In highly criminalized settings KP may be more vulnerable to delay or not to seek HIV services. Without such support for safe linkage to HIV services, HIVST may be of limited benefit to KP in such settings.

We found no clear evidence to support adverse events as a result of HIVST, such as adverse emotional reactions to positive tests, inter-partner violence, coerced/forced testing, psycho-social or mental health issues, and suicide or self-harm. This is in line with a recent literature review which states that very few studies report harm across various self-tests, including HIV; however it does note that monitoring and reporting systems for harmful outcomes are rare [61].

## Limitations

The majority of studies that met inclusion criteria were among MSM and in HIC settings. Only two studies provided data on user preferences among MSM and FSW [31, 35]. Our search was for KP, however due to the nature of self-testing, people in prison or closed settings, would not be eligible for HIVST. Almost all studies were observational and used a cross-sectional research design. Only one study in this review was a randomized control trial. We cannot therefore rule out selection bias, including sample representativeness and non-response rate. The inclusion criteria for this review were overly inclusive to capture all or any values and preferences on HIVST among KP. Therefore, study designs, characteristics and sample sizes were heterogeneous, and results may not be generalizable.

Most studies had incomplete reporting of data items and low compliance with the STROBE reporting checklist.

## Conclusion

MSM in HIC settings find oral fluid-based HIVST to be highly acceptable, using a supervised or an unsupervised approach. However, concerns about counseling, user error and poor accuracy remain. Data on social harm and adverse events resulting from HIVST was not reported. To better understand user concerns, as well as risk of adverse event and potential social harm, rigorous monitoring and reporting systems should be implemented, so that program managers and policy-makers can consider the potential risks and benefits for introducing HIVST among KP.

The convenience and private nature of HIVST is reportedly advantageous to MSM, and may also be so for other KP, including SW, PWID and transgender people in HIC, MIC or LIC settings. However, information among KP, other than MSM, and in low- or middle-income settings is limited.

Key population values and preferences around HIVST should be considered by researchers, policy-makers and program managers, as HIVST may be an additional approach to reverse inequities in access to HIV testing for KP who have a low access to HIV services, but carry much of the HIV burden globally. Taking into account our study results, more data from diverse settings and among non-MSM key population groups is needed to better understand the potential impact of self-testing as part of the global HIV response.

## Electronic supplementary material

Supplementary material 1 (DOCX 44 kb)
