# Supplementary material for: Attitudes and Acceptability on HIV Self-testing Among Key Populations: A Literature Review
Source: AIDS Behav. 2015 Jun 9;19(11):1949–65. doi: 10.1007/s10461-015-1097-8 (PMC4598350; doi:10.1007/s10461-015-1097-8)
Supplement: Supplementary file 1 — Supplementary material 1 (DOCX 44 kb) [file 10461_2015_1097_MOESM1_ESM.docx]

**Table S1: STROBE reporting criteria for cross-sectional studies (full-text)**

| **STROBE Recommendation**  **(For cross-sectional studies – full-text)** | **De La Fuente 2012 [39]** | **Carballo-Dieguez 2012[17]** | **Gray 2013 [34]** | **Lee 2007 [42]** | **Ng 2012 [44]** | **Skolnik 2001 [45]** | **Xun 2013 [47]** | **Lippman 2014 [43]** | **Marley 2014 [35]** | **Spielberg 2003 [46]** | **Carballo-Dieguez 2012 [33]** | **Greacen 2013 [40]** | **MiraTes 2007 [30]** | **Chen 2010 [38]** | **Ochako 2014 [31]** | **Han 2014 [41]** | **Bavinton 2013 [15]** |
| --- | --- | --- | --- | --- | --- | --- | --- | --- | --- | --- | --- | --- | --- | --- | --- | --- | --- |
| 1. Indicate the study’s design with a commonly used term in the title or the abstract | NR | R | R | R | R | NR | R | R | R | R | NR | R | NR | R | R | R | R |
| 1. Provide in the abstract an informative and balanced summary of what was done and what was found | R | R | R | R | R | R | R | R | R | R | R | R | NR | R | R | R | R |
| 1. Explain the scientific background and rationale for the investigation being reported | R | R | R | R | R | R | R | R | R | R | R | R | R | R | R | R | R |
| 1. State specific objectives, including any pre-specified hypotheses | R | R | R | R | R | R | R | R | R | R | R | R | R | R | R | R | R |
| 1. Present key elements of study design early in the paper | R | R | R | R | R | R | R | R | R | R | R | R | R | R | R | R | R |
| 1. Describe the setting, locations, and relevant dates, including periods of recruitment, exposure, follow-up, and data collection | R | R | R | R | R | R | R | R | R | R | NR | R | R | R | R | R | NR |
| 1. Give the eligibility criteria, and the sources and methods of selection of participants | R | R | R | R | R | R | R | R | R | R | R | R | R | R | R | R | R |
| 1. Clearly define all outcomes, exposures, predictors, potential confounders, and effect modifiers. Give diagnostic criteria, if applicable | R | R | R | R | R | R | R | R | R | R | R | R | R | R | R | R | R |
| 1. For each variable of interest, give sources of data and details of methods of assessment (measurement). Describe comparability of assessment methods if there is more than one group | R | R | R | R | R | R | R | R | R | R | R | R | R | R | R | R | R |
| 1. Describe any efforts to address potential sources of bias | NR | NR | NR | NR | R | NR | NR | NR | NR | NR | R | NR | NR | NR | NR | NR | NR |
| 1. Explain how the study size was arrived at | NR | NR | NR | NR | NR | R | R | R | R | R | R | R | NR | R | R | R | R |
| 1. Explain how quantitative variables were handled in the analyses. If applicable, describe which groupings were chosen and why | R | R | NR | R | R | NR | R | NR | NR | R | NR | NR | NR | NR | R | NR | R |
| 1. Describe all statistical methods, including those used to control for confounding | NR | R | R | R | R | NR | R | R | R | R | NR | R | NR | R | R | R | R |
| 1. Describe any methods used to examine subgroups and interactions | NR | NR | NR | R | NR | NR | NR | NR | NR | NR | NR | NR | NR | NR | NR | NR | R |
| 1. Explain how missing data were addressed | NR | NR | NR | NR | NR | NR | NR | NR | NR | NR | NR | NR | NR | NR | NR | NR | NR |
| 1. If applicable, describe analytical methods taking account of sampling strategy | NR | NR | R | NR | NR | NR | NR | NR | NR | NR | NR | NR | NR | NR | NR | NR | NR |
| 1. Describe any sensitivity analyses | NR | NR | NR | NR | NR | NR | NR | NR | R | NR | NR | NR | NR | NR | NR | NR | NR |
| 1. Report numbers of individuals at each stage of study—eg numbers potentially eligible, examined for eligibility, confirmed eligible, included in the study, completing follow-up, and analyzed | NR | R | NR | R | R | R | R | R | R | R | R | R | R | R | R | R | NR |
| 1. Give reasons for non-participation at each stage | NR | R | NR | R | NR | NR | R | R | R | NR | R | R | NR | R | R | R | R |
| 1. Consider use of a flow diagram | R | NR | NR | NR | NR | NR | NR | NR | NR | NR | NR | NR | NR | NR | NR | NR | NR |
| 1. Give characteristics of study participants (e.g. demographic, clinical, social) and information on exposures and potential confounders | R | R | R | R | R | R | R | R | NR | R | R | R | R | R | R | R | R |
| 1. Indicate number of participants with missing data for each variable of interest | R | NR | NR | NR | NR | NR | NR | NR | NR | NR | NR | NR | NR | NR | R | NR | NR |
| 1. Report numbers of outcome events or summary measures | R | NR | R | R | R | R | R | R | R | R | R | R | R | R | R | R | R |
| 1. Give unadjusted estimates and, if applicable, confounder-adjusted estimates and their precision (e.g., 95% confidence interval). Make clear which confounders were adjusted for and why they were included | R | NR | NR | R | R | R | NR | NR | NR | NR | NR | R | NR | NR | NR | R | NR |
| 1. Report category boundaries when continuous variables were categorized | NR | NR | NR | NR | NR | NR | R | NR | NR | NR | NR | NR | R | NR | R | R | NR |
| 1. If relevant, consider translating estimates of relative risk into absolute risk for a meaningful time period | NR | NR | NR | NR | NR | NR | NR | NR | NR | NR | NR | NR | NR | NR | NR | NR | NR |
| 1. Report other analyses done—eg analyses of subgroups and interactions, and sensitivity analyses | NR | NR | R | NR | NR | NR | NR | NR | R | NR | NR | NR | NR | NR | NR | NR | NR |
| 1. Summarize key results with reference to study objectives | R | R | R | R | R | R | R | R | R | R | R | R | R | R | R | R | R |
| 1. Discuss limitations of the study, taking into account sources of potential bias or imprecision. Discuss both direction and magnitude of any potential bias | R | R | R | R | R | R | R | R | R | R | R | R | NR | R | R | R | R |
| 1. Give a cautious overall interpretation of results considering objectives, limitations, multiplicity of analyses, results from similar studies, and other relevant evidence | R | R | R | R | R | R | R | R | R | R | R | NR | R | R | R | R | R |
| 1. Discuss the generalizability (external validity) of the study results | NR | R | R | R | R | R | NR | R | R | R | R | NR | R | NR | R | NR | R |
| 1. Give the source of funding and the role of the funders for the present study and, if applicable, for the original study on which the present article is based | R | R | R | R | R | R | R | R | R | R | R | R | R | NR | R | R | R |

R-reported, NR not reported.

**Table S2: STROBE reporting criteria for cross-sectional studies (conference abstracts)**

| **STROBE Recommendations**  **(For conference abstracts)** | **Bavinton 2014 [48]** | **Wong 2014 [51]** |
| --- | --- | --- |
| 1. Indicate the study’s design with a commonly used term in the title | NR | NR |
| 1. Contact details for the corresponding author | R | R |
| 1. Description of the study design | R | R |
| 1. Specific objectives or hypothesis | R | R |
| 1. Description of setting, follow-up dates or dates at which the outcome events occurred or at which the outcomes were present, as well as any points or ranges on other time scales for the outcomes | R | R |
| 1. Give the eligibility criteria, and the major sources and methods of selection of participants | NR | R |
| 1. Clearly define primary outcome for this report. | R | R |
| 1. Describe statistical methods, including those used to control for confounding | NR | NR |
| 1. Report Number of participants at the beginning and end of the study | NR | R |
| 1. Report estimates of associations. If relevant, consider translating estimates of relative risk into absolute risk for a meaningful time period Report appropriate measures of variability and uncertainty (e.g., odds ratios with confidence intervals | NR | NR |
| 1. General interpretation of study results | R | R |

R-reported, NR not reported.

**Table S3: STROBE reporting criteria for cohort studies (conference abstracts)**

| **STROBE Recommendations**  **(For conference abstracts)** | **Chakravarty 2014 [49]** | **Mayer 2014 [50]** |
| --- | --- | --- |
| 1. Indicate the study’s design with a commonly used term in the title | NR | NR |
| 1. Contact details for the corresponding author | NR | R |
| 1. Description of the study design | R | NR |
| 1. Specific objectives or hypothesis | R | NR |
| 1. Description of setting, follow-up dates or dates at which the outcome events occurred or at which the outcomes were present, as well as any points or ranges on other time scales for the outcomes | R | NR |
| 1. Give the eligibility criteria, and the major sources and methods of selection of participants | NR | NR |
| 1. Clearly define primary outcome for this report. | R | R |
| 1. Describe statistical methods, including those used to control for confounding | NR | NR |
| 1. Report Number of participants at the beginning and end of the study | NR | R |
| 1. Report estimates of associations. If relevant, consider translating estimates of relative risk into absolute risk for a meaningful time period Report appropriate measures of variability and uncertainty (e.g., odds ratios with confidence intervals | NR | NR |
| 1. General interpretation of study results | R | R |

R-reported, NR not reported.

**Table S4: CONSORT reporting criteria for RCT (conference abstract)**

| **CONSORT Recommendations**  **(For conference abstracts)** | **Katz 2012**  **[16]** |
| --- | --- |
| 1. Identification of the study as randomized in the title | NR |
| 1. Contact details for the corresponding author | R |
| 1. Description of the trial design (e.g. parallel, cluster, non-inferiority) | R |
| 1. Eligibility criteria for participants and the settings where the data were collected | R |
| 1. Interventions intended for each group | R |
| 1. Specific objective or hypothesis | R |
| 1. Clearly defined primary outcome for this report | R |
| 1. How participants were allocated to interventions | NR |
| 1. Whether or not participants, care givers, and those assessing the outcomes were blinded to group assignment | R |
| 1. Number of participants randomized to each group | NR |
| 1. Trial status | NR |
| 1. Number of participants analysed in each group | NR |
| 1. For the primary outcome, a result for each group and the estimated effect size and its precision | R |
| 1. Important adverse events or side effects | R |
| 1. General interpretation of the results | R |
| 1. Registration number and name of trial register | NR |
| 1. Source of funding | NR |

R-reported, NR not reported.


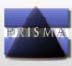
**PRISMA 2009 Checklist**

| **Section/topic** | **#** | **Checklist item** | **Reported on page #** |
| --- | --- | --- | --- |
| **TITLE** | | |  |
| Title | 1 | Identify the report as a systematic review, meta-analysis, or both. | 1 |
| **ABSTRACT** | | |  |
| Structured summary | 2 | Provide a structured summary including, as applicable: background; objectives; data sources; study eligibility criteria, participants, and interventions; study appraisal and synthesis methods; results; limitations; conclusions and implications of key findings; systematic review registration number. | 1 |
| **INTRODUCTION** | | |  |
| Rationale | 3 | Describe the rationale for the review in the context of what is already known. | 3-5 |
| Objectives | 4 | Provide an explicit statement of questions being addressed with reference to participants, interventions, comparisons, outcomes, and study design (PICOS). | 4-5 |
| **METHODS** | | |  |
| Protocol and registration | 5 | Indicate if a review protocol exists, if and where it can be accessed (e.g., Web address), and, if available, provide registration information including registration number. | N/A |
| Eligibility criteria | 6 | Specify study characteristics (e.g., PICOS, length of follow-up) and report characteristics (e.g., years considered, language, publication status) used as criteria for eligibility, giving rationale. | 5-6 |
| Information sources | 7 | Describe all information sources (e.g., databases with dates of coverage, contact with study authors to identify additional studies) in the search and date last searched. | 5-6 |
| Search | 8 | Present full electronic search strategy for at least one database, including any limits used, such that it could be repeated. | N/A |
| Study selection | 9 | State the process for selecting studies (i.e., screening, eligibility, included in systematic review, and, if applicable, included in the meta-analysis). | 5-6 |
| Data collection process | 10 | Describe method of data extraction from reports (e.g., piloted forms, independently, in duplicate) and any processes for obtaining and confirming data from investigators. | 5-6 |
| Data items | 11 | List and define all variables for which data were sought (e.g., PICOS, funding sources) and any assumptions and simplifications made. | 6-7 |
| Risk of bias in individual studies | 12 | Describe methods used for assessing risk of bias of individual studies (including specification of whether this was done at the study or outcome level), and how this information is to be used in any data synthesis. | N/A |
| Summary measures | 13 | State the principal summary measures (e.g., risk ratio, difference in means). | N/A |
| Synthesis of results | 14 | Describe the methods of handling data and combining results of studies, if done, including measures of consistency (e.g., I^2^) for each meta-analysis. | 6-7 |
| Risk of bias across studies | 15 | Specify any assessment of risk of bias that may affect the cumulative evidence (e.g., publication bias, selective reporting within studies). | N/A |
| Additional analyses | 16 | Describe methods of additional analyses (e.g., sensitivity or subgroup analyses, meta-regression), if done, indicating which were pre-specified. | N/A |
| **RESULTS** | | |  |
| Study selection | 17 | Give numbers of studies screened, assessed for eligibility, and included in the review, with reasons for exclusions at each stage, ideally with a flow diagram. | 8-9 |
| Study characteristics | 18 | For each study, present characteristics for which data were extracted (e.g., study size, PICOS, follow-up period) and provide the citations. | 8-9 |
| Risk of bias within studies | 19 | Present data on risk of bias of each study and, if available, any outcome level assessment (see item 12). | N/A |
| Results of individual studies | 20 | For all outcomes considered (benefits or harms), present, for each study: (a) simple summary data for each intervention group (b) effect estimates and confidence intervals, ideally with a forest plot. | N/A |
| Synthesis of results | 21 | Present results of each meta-analysis done, including confidence intervals and measures of consistency. | N/A |
| Risk of bias across studies | 22 | Present results of any assessment of risk of bias across studies (see Item 15). | N/A |
| Additional analysis | 23 | Give results of additional analyses, if done (e.g., sensitivity or subgroup analyses, meta-regression [see Item 16]). | N/A |
| **DISCUSSION** | | |  |
| Summary of evidence | 24 | Summarize the main findings including the strength of evidence for each main outcome; consider their relevance to key groups (e.g., healthcare providers, users, and policy makers). | 14-17 |
| Limitations | 25 | Discuss limitations at study and outcome level (e.g., risk of bias), and at review-level (e.g., incomplete retrieval of identified research, reporting bias). | 18 |
| Conclusions | 26 | Provide a general interpretation of the results in the context of other evidence, and implications for future research. | 19 |
| **FUNDING** | | |  |
| Funding | 27 | Describe sources of funding for the systematic review and other support (e.g., supply of data); role of funders for the systematic review. | 19 |

Page 1 of 2

*From:*  Moher D, Liberati A, Tetzlaff J, Altman DG, The PRISMA Group (2009). Preferred Reporting Items for Systematic Reviews and Meta-Analyses: The PRISMA Statement. PLoS Med 6(6): e1000097. doi:10.1371/journal.pmed1000097

For more information, visit: **www.prisma-statement.org**.

Page 2 of 2
